# ‘Waiting for’ and ‘waiting in’ public and private hospitals: a qualitative study of patient trust in South Australia

**DOI:** 10.1186/s12913-017-2281-5

**Published:** 2017-05-05

**Authors:** Paul R. Ward, Philippa Rokkas, Clinton Cenko, Mariastella Pulvirenti, Nicola Dean, A. Simon Carney, Samantha Meyer

**Affiliations:** 1 Discipline of Public Health, Flinders University, Room 2.10, level 2, Health Science Building, Registry Road, Bedford Park, 5042 SA Australia; 2 Flinders Medical Centre, Adelaide, Australia; 3 School of Medicine, Flinders University, Adelaide, Australia; 4 School of Public Health and Health Systems, University of Waterloo, Waterloo, Canada

**Keywords:** Waiting times, Trust, Public hospitals, Private hospitals, Qualitative, Australia

## Abstract

**Background:**

Waiting times for hospital appointments, treatment and/or surgery have become a major political and health service problem, leading to national maximum waiting times and policies to reduce waiting times. Quantitative studies have documented waiting times for various types of surgery and longer waiting times in public vs private hospitals. However, very little qualitative research has explored patient experiences of waiting, how this compares between public and private hospitals, and the implications for trust in hospitals and healthcare professionals. The aim of this paper is to provide a deep understanding of the impact of waiting times on patient trust in public and private hospitals.

**Methods:**

A qualitative study in South Australia, including 36 in-depth interviews (18 from public and 18 from private hospitals). Data collection occurred in 2012–13, and data were analysed using pre-coding, followed by conceptual and theoretical categorisation.

**Results:**

Participants differentiated between experiences of ‘waiting for’ (e.g. for specialist appointments and surgery) and ‘waiting in’ (e.g. in emergency departments and outpatient clinics) public and private hospitals. Whilst ‘waiting for’ public hospitals was longer than private hospitals, this was often justified and accepted by public patients (e.g. due to reduced government funding), therefore it did not lead to distrust of public hospitals. Private patients had shorter ‘waiting for’ hospital services, increasing their trust in private hospitals and distrust of public hospitals. Public patients also recounted many experiences of longer ‘waiting in’ public hospitals, leading to frustration and anxiety, although they rarely blamed or distrusted the doctors or nurses, instead blaming an underfunded system and over-worked staff. Doctors and nurses were seen to be doing their best, and therefore trustworthy.

**Conclusion:**

Although public patients experienced longer ‘waiting for’ and ‘waiting in’ public hospitals, it did not lead to widespread distrust in public hospitals or healthcare professionals. Private patients recounted largely positive stories of reduced ‘waiting for’ and ‘waiting in’ private hospitals, and generally distrusted public hospitals. The continuing trust by public patients in the face of negative experiences may be understood as a form of *exchange trust norm*, in which institutional trust is based on base-level expectations of consistency and minimum standards of care and safety. The institutional trust by private patients may be understood as a form of *communal trust norm*, whereby trust is based on the additional and higher-level expectations of flexibility, reduced waiting and more time with healthcare professionals.

## Background

Australia has a mixed-system of healthcare – a publically funded system (Medicare) alongside private healthcare funded through Private Health Insurance (PHI) and direct fees. Whilst this funds lots of non-hospital services, this paper focuses specifically on public and private hospitals. Public hospital treatment is free to ‘public patients’ (i.e. those people without PHI). However, ‘private patients’ can be treated in either public or private hospitals, both situations paid for through their private health insurer. Although waiting times are generally accepted as a ‘fact of life’ in publically-funded healthcare systems [1], and a perceived inevitable bi-product of an over-burdened health system, there is negligible qualitative research on the impact of ‘waiting’ on patient trust in public vs. private hospitals. There have been a number of qualitative studies of patient experiences of waiting for various types of surgery [1–4], but none of these studies explicitly compared the experiences of waiting in public and private systems and they did not explore the ways in which waiting links to trust or distrust in providers and/or hospitals. Nevertheless, one study [3] did make a link between trust and waiting, finding that “establishing a trusting relationship with health-care representatives can help the person endure the wait for surgery more easily” (p. 534).

Trust is fundamental to health care consumers’ capacity to secure a positive encounter with the health system and its representatives. The ability to trust the healthcare system is essential for consumers to make informed decisions regarding their health [5, 6]. Changes in health care financing, organization and technology, as well as changes to doctor-patient relationships have increased interest in the concept of patient trust in the past decade [7–10]. Trust has therefore become a prominent healthcare issue among patients and doctors, as well as among authorities, policy-makers and the public in general [11]. It is integral to both the patient experiences, attitudes and behaviours, and consequently, patient health outcomes [12].

The physical and psychological impact of waiting for medical treatment either before, during or after, a medical encounter is of great interest in health services research and healthcare spending [3, 4, 13]. Longer waiting times have been associated with longer mean length of stay and greater short-term risk of death [1]. While many aspects of hospitals are under public scrutiny, such as access to hospital services, the quality of care, funding and management arrangements [14], literature regarding the actual impact of consumer trust while waiting for medical care in the Australian health system is negligible. This is surprising considering the strategic and often emotive political, socio-economic significance placed by society on hospital waiting times. Overwhelmingly, discussions about healthcare in Australia focus on the performance of public sector hospitals, and waiting times often forms an important component of the assessment of performance. This is reflected in the establishment of hospital key performance indicators related to waiting times for the provision of emergency hospital care [15]. There are questions left unanswered regarding what these quantified waiting times, reported as efficiency measures, may mean for Australian health care consumers and whether or not waiting for medical care impacts their trust of the health system.

Within this paper, we draw on interviews with patients, whereby they talked about the various ways in which *waiting* impacted their trust in both healthcare professionals (interpersonal trust) and various institutions such as public and private hospitals and governments (institutional trust). Interpersonal trust has been defined as ‘the mutual confidence that no party to an exchange will exploit the other’s vulnerability’[16] (p. 1133), with a trustworthy healthcare professional having both good intentions and reasonable competence [17]. A definition of institutional trust is ‘the *expected* [emphasis added] utility of institutions performing satisfactorily’ [18]. In our case, this may depend on the patient having experience with the hospital, otherwise they may not be able to say “one way or the other whether they [institutions] are trustworthy” [19], since “trust arises when a community shares a set of moral values in such a way as to create expectations of regular and honest behaviour” [20] (p.153).

Within the interviews that form the basis for this paper, participants spoke differently about their trust in various parts of government, hospitals and different healthcare professionals. However, we cannot fully dissect their trust in hospitals from their trust in healthcare professionals, since they interpersonal and institutional trust relate to and impact on each other [8, 21–24]. The complexities of studying *either* institutional *or* interpersonal trust have been articulated by Giddens in terms of the dualism of trust [25] which argues for the inter-connected and bi-directional nature of trust – trust in one impacts on trust in the other, and vice versa. Indeed, Govier said that, “When we assume that an object [in this context, health care] will serve its function, we are, in effect, assuming that the various people who manufactured and marketed it did their jobs honestly and properly. If these objects do not perform, someone somewhere made a mistake” [17] (p. 16). On this basis, for patients to trust (or distrust) the hospital (as the object), they need to also have some level of trust in the healthcare professionals working there, the government funding it, the professional organisations supporting it and so on. In this paper, we examine how waiting impacts patient trust in both public and private hospitals and the healthcare professionals working within them.

### Waiting times in public and private healthcare systems

Research investigating trust in Australian healthcare suggests that purchasing PHI is driven by a set of culturally informed expectations about trust in the Australian public health care system [26]. Subsequent research has identified that private health insurance holders are fearful and indeed lack trust in public hospitals, the drivers of which are also largely culturally informed expectations [27]. These cultural expectations unmet by the public system are likely because PHI holders are privy to being assigned higher urgency, ‘jump the queues’ for procedures and are provided more medical and diagnostic procedures, irrespective of healthcare needs [28]. Indeed, a study on reasons for purchasing PHI in Australia identified wanting reduced waiting times and increased trust in private healthcare (in a broad sense) as important, although they did not interrogate the predictors, extent or reasons in detail [26]. In both aforementioned studies, waiting times are stated as a driver for both purchasing PHI, and lower trust in public healthcare.

Public hospital patients however have been identified in a previous paper by the authors as expressing “pragmatic acceptance” (i.e. recognising the problem of wait times is intractable) of the ‘failures’ in publicly funded hospitals and therefore, were unwilling to criticise or challenge either their doctors or the healthcare system [29]. This finding is echoed in research out of the UK whereby public patients expressed a ‘will to trust’, leading researchers to suggest that excusing the identified faults of the system can be therapeutic and reduces anxiety [30]. These data are suggestive that perceptions of public healthcare differ between those with PHI, and those without. How this impacts on the nature (or extent) of patient trust remains equivocal.

Public hospitals are fundamental to the structure and working of Australia’s public health system; they are the primary source of hospital services for the majority of Australians [31]. In 2013, 53% of the Australian population did not have PHI [32]. The fact that publically-funded health care systems have waiting times for surgical procedures is not new, and is simply a factor of greater demand than supply [33]. In resource-limited, taxation-funded settings, there are more patients in need of surgery than resources available to provide the surgery to those patients at that time, simply because governments need to allocate funding across all public services.

A number of countries, including Australia, have attempted various measures to reduce waiting times and introduced national maximum waiting times for surgery [34]. The consensus across international literature on waiting times is that waiting times need to be reduced in order to improve health [13, 33, 34]. Nevertheless, waiting times for surgery in private hospitals are much shorter than in public hospitals [33] and waiting times over 12 months for surgery in public hospitals is an independent predictor of increasing demand for PHI [13]. Long waiting times, or at least the perception of long waiting times, for surgery in public hospitals encourages people to pay for PHI (alongside other factors, including choice over doctor, aesthetics of infrastructure, and government rebates [26, 27]), due to the reduced waiting times in the private system. Recent research on international comparisons of waiting times between countries has attempted to make distinctions between various ‘waits’ in the public system [33]. This research highlights the different waiting times between the initial general practitioner referral and surgery, between the general practitioner referral and seeing the hospital specialist, and also between seeing the hospital specialist and finally having the surgery [33, 34]. These different waiting times in previous studies have been presented using quantitative data, although they fail to uncover the different experiences of waiting.

## Methods

Our previous paper based on the same interviews provides more detail on the methods and analysis [29], although we provide a brief overview here. A qualitative methodology was used to explore the experiences, perceptions and observations of patients who had recently been treated within either public and/or private hospitals. Our focus in the interviews was on understanding patient trust in public and private hospitals. Participants were sampled using a purposeful sampling method. The literature suggests that riskier medical procedures have different trust dynamics [35, 36], thus it was important to recruit patients who had experienced various levels of risk during their treatment. The purposive sampling was based on aiming to interview a mix of patients who were undergoing urgent, semi-urgent and non-urgent procedures (this is the terminology used in hospitals), in both private and public hospitals, so that we could get a broad range of perspectives. Participants were initially recruited from both Plastic and Reconstructive Surgery and Ear, Nose and Throat clinics in public and private hospitals in South Australia. These patients had prior experience of these hospitals, although we could not access data on exactly how long their waits had been to access surgery prior to the interviews. Nevertheless, during interviews, they talked at length about various experiences of waiting for different types of health care for different severity of health problems, adding to the breadth of experiences they could draw from.

Recruitment included four different methods. First, three consultant surgeons from both private and public clinics distributed information packages to eligible patients. This recruitment method did not yield enough participants (*n =* 6), so we instituted a second approach. The research assistant sat in the waiting room of two of the surgical clinics and distributed information packages to potential participants, which resulted in a further seven. Third, we placed an advertisement in the e-bulletin of the Health Consumer’s Alliance of SA Inc., which resulted in three more participants. Finally, an advertisement was placed in the electronic newsletter of a local university, resulting in a further 20 more participants. In this final recruitment, we recruited people who had personal experience of surgery in either of the public or private hospitals in South Australia, but not necessarily the Plastic and Reconstructive Surgery and Ear, Nose and Throat clinics. Whilst this could potentially have introduced a slightly different kind of participant, during the interviews, participants talked about numerous experiences of waiting for and care in multiple hospitals, and it added breadth to the final sample.

The 36 participants (12 males and 24 females) ranged from 25 to 87 years in age, and included 18 participants from both public and private hospitals, with an even spread between the two types of surgical units. The gender imbalance in our final sample may mean that our findings need to be interpreted with care, although during analysis, we did not identify particular differences in between men and women in their perceptions of waiting times or trust in public or private hospitals. Written consent was obtained prior to the interview. In-depth interviews were conducted between 2012–13, at a mutually convenient time at the participant’s home, or a location of their choosing. Interviews were approximately one hour in length. The interviews explored their actual experiences of being patients in hospitals, although they often also talked about their experiences of caring for friends/family in hospital. Whilst this was not technically their perceptions as a patient, the additional experience shaped their expectations of and trust in hospitals. In all interviews, the researcher (CC) probed for more detail on their perceptions of the care they received, their expectations of care, and whether these were met. This nuanced and contextually contingent approach allowed us to more fully understand their narratives, as opposed to simply asking whether or not they trust their doctor, which may have just led to stereotypical factoids [37]. Interviews were audio taped and transcribed verbatim for the purpose of analysis using NVivo 8 software. Each interview was transcribed directly after the interview so that the data analysis and collection could be compared.

Three stages of analysis were undertaken: pre-coding, conceptual categorisation and theoretical categorisation, explained in more detail elsewhere [29]. The process of pre-coding consisted of identifying words most frequently used by the participants in interviews. When the data were pre-coded, words, or sections of text, were coded using the actual words used by participants or by grouping similar words conceptually. This process was undertaken throughout the data collection process, and the initial pre-coding informed the content of subsequent interviews. Pre-coding was undertaken separately by three of the authors (CC, PW & SM) and discussion of coding and further refining was undertaken. Conceptual categorisation was undertaken by grouping the initial codes into larger categories. This process (mainly by CC and PR) involved an iterative process of inserting each of the initial codes into larger categories, based on their ‘semantic fit’ or the ways in which they seemed to be relating to a similar idea or issue. Theoretical categorisation was conducted (mainly by PW & SM) by examining the focused codes with regards to theoretical and empirical literature on trust, it highlighted data that both conformed to current theories of trust and also yielded ‘new data’. In particular, we assessed differences and similarities between participants with and without PHI in terms of their experiences of waiting and attitudes towards public and private hospitals. Frequent discussions with all of the research team occurred to validate emerging codes.

## Results

We present data from patients who drew on their experiences (rather than quantifying length) of waiting times, and the impact this had on their perception of hospital services and staff. Our participants talked differently about ‘waiting for’ hospital appointments and surgery once referred, and ‘waiting in’ hospitals (e.g. waiting in emergency departments, waiting to see healthcare professionals once admitted) and how this differed between public and private hospitals. Participant’s perceptions of the reasons for and acceptance of ‘waiting for’ or ‘waiting in’ hospitals impacted their trust in various social systems (e.g. government, hospitals) and healthcare professionals working within hospitals. Although participants’ discussions of ‘waiting for’ and ‘waiting in’ hospitals may not map easily onto administrative definitions [4], we provide contextual data to understand the impact of waiting times on patient experiences. It is important to provide this rich, contextual and meaningful data on the lived experiences of waiting in public and private hospitals, since “knowing more about what conditions produce trust and distrust, and why this matters, helps to craft the structure and financing of health care delivery in a manner that supports and enhances trust” [38]. This may provide a more comprehensive picture of how waiting time shapes trust and consequential attitudes and health behaviours relevant to healthcare expenditure.

### Differences in the experiences of ‘waiting for’ and ‘waiting in’ public and private hospitals

All participants made stark comparisons in terms of ‘waiting for’ and ‘waiting in’ public and private hospitals. The perceptions outlined in this section did not vary on the basis of the urgency of the treatment or surgery required by participants. Participants in public hospitals had experienced much longer waiting times for hospital appointments and for elective surgery. The main reason for most participants not purchasing PHI was the cost, and most would have liked to for the purpose of reducing waiting for treatment, but could not afford to. For example, HH said that she would consider taking out PHI to *“avoid a hideously long waiting list*” and that “*we would then just make arrangements to pay for private options and then cop it sweet*”. The term ‘cop it sweet’ suggests that HH would accept the financial consequences in terms of potentially not being able to afford other things in life as a result of paying for PHI. The main reason for choosing and paying for PHI was to reduce ‘waiting for’ times, as SC said, *“I don’t want to hang around. I want action and I want it now. You’re not going to get that if you don’t have private health cover, otherwise you go on the waiting list”.* Both of these participants talked about wanting to reduce the times ‘waiting for’ hospital care, and suggested that PHI would provide this for them – an indicator of trust in PHI, compared to the public system, to reduce times ‘waiting for’ hospital care.

One participant (SC) who had experienced care in both public and private hospitals described an incident waiting in a public hospital when she was left alone before a surgical procedure. Her remarks throughout her interview suggest that this experience decreased her trust in the public hospital system, not directly because of the quality of care, but because of the waiting and feelings of isolation. Similar comments were made by most public patients in this study, who rarely cited the quality of clinical care as their reasons for reduced trust, but were concerned about the lack of attention given to them while in hospital, making them question their trust in the system as opposed to the individual doctors and nurses. SC kept referring back to the ‘waiting in’ public hospital incident throughout the interview. Although she defended the doctors, her repeated references to waiting indicated that it had become an issue for her and had made her anxious regarding future hospital visits. She compared this experience to a similar situation of ‘waiting in’ a private hospital with a more favourable outcome, where she was checked on every hour and thus not left alone. Hence, this made the waiting manageable. The lack of contact and isolation is perhaps the key to her negative experience and loss of trust in public hospitals:

*“..the fact that I got left, I suppose, is my main issue, I mean I was put in that room at six o’clock in the morning and I did not see anyone until I rang a bell at two o’clock in the afternoon because I had a throbbing headache from not eating, all day… It would have been nice to like – I don’t know, my experience in the private system was every hour or so they used to come in and say ‘how you going? You all right?’ You know, such and such will let you know and ‘it’s another few hours until you’ll be seen’ but there, they just left me and I never saw anybody until I rang this bell”* (SC)

SC, along with many other participants, talked about how the isolation, feeling ‘forgotten’ and being bereft of information left them feeling less trusting of public hospitals. SC talked about how her previous experience makes her feel about future encounters in public hospitals:

*“probably a little bit less confident because I am going in to have surgery again soon for a totally unrelated issue to this in July and I am a bit concerned because that’s through the public system again. I am a bit concerned whether – because this is a bit more major - whether I’m going to be treated like I was then and just left in a room to fend for myself or whether this is going to occur again so, yeah, it has got me a bit wary”* (SC).

Another participant (JI) described his young daughter’s ‘*long’* and ‘*traumatic*’ experience ‘waiting in’ a public hospital for surgery, and articulated how he would have been ‘*prepared* ‘if he had just had some information:

*I’d be better prepared and I’d make sure I’d got heaps of books for the kids and I’d just prepare them for the fact that they may be waiting for hours and hours”* (JI).

On reflection of his daughter’s experience, JI identified the fault of the hospital, suggesting that the hospital practices could be improved: “*I just think it could have been done more efficiently.”* He favourably contrasted his daughter’s ‘waiting in’ private hospital experience to that in the public system, observing that it was, ‘streamlined,’ ‘efficient,’ and the nurses, ‘*come out and tell you how it was going. It was a much more pleasant experience’* (JI).

For all participants, there was a general perception that nurses in public hospitals were busier, *“they don’t have time to chat”* (MT), than the nurses in private hospitals, who were perceived as having more time to spend with patients, thereby improving the quality of care and reducing ‘waiting in’ the hospital:

*“If you’re a patient in hospital and you need to see a nurse you don’t want to be waiting or you don’t think you want to be waiting. The nurses in the private sector are probably looking after less people than the nurses in the public….Probably not any better than the nurse in the public sector could but certainly quicker because they haven’t got as much to do I don’t think. If you ring your bell for a nurse to come if you can’t get out of bed and it takes half an hour you’re worrying about it. If the nurse can come within five minutes it’s less stressful”* (GW).

This quote highlights GWs identification that nurses are ‘busier’ in public hospitals, but not necessarily providing poorer quality care and therefore not less trustworthy. In contrast to feeling isolated and forgotten while ‘waiting in’ public hospitals, a number of participants from public hospitals made assumptions about what it would be like to be treated in a private hospital, drawing on cultural expectations regarding privatisation. All public hospital participants assumed that both ‘waiting for’ and ‘waiting in’ would be shorter in the private system, that the hospitals would look nicer but interestingly that patients would be made to feel more ‘special’ in private hospitals. Although not explicit in the following quote, a question for future research relates to the extent to which such feelings of being ‘special’ impact positively on trust:

*“I don’t think the quality of an operation would make a difference at all, I think it just may be longer queues in the public sector. Maybe also people like to feel, you know, as a private patient I guess maybe you’re made to feel more special; I don’t know and I haven’t been in the private care so I couldn’t really say”* (MT)*.*

Although participants with PHI talked about shorter ‘waiting in’ times and perceived superior service in private hospitals, this did not translate into perceptions of better clinical care. LK describes a common theme about the ‘service’ (e.g. speediness of service, friendliness of staff, time spent with health care professionals) being better but not necessarily the quality of clinical care. In this way, there is a level of trust in the efficiency of private hospitals but this does not necessarily lead to either an increase in trust in clinical care in private, or reduction in public, hospitals:

*“Health care wise I don’t think so. I think you get better service. You get through straightaway sort of thing, you know? I mean private health cover is useful when you want elective operations or surgeries. I think the service is better but the health care, health care wise, not necessarily so”* (LK).

Participants were reluctant to denigrate the care they received in public hospitals, irrespective of the urgency of their treatment or the length of their wait, but often followed positive comments (seemingly not wanting to appear distrusting of public hospitals) about the system with quasi-apologetic comments regarding services that they considered missing, such as information:

*“Oh yeah, absolutely and the medical care was the best. I really – they couldn’t have done much more different with the medical care; that was all good. It was just the lack of information and again I found that the same with my daughter. You know, knowing what services were available, especially since I don’t have family in Australia, all this kind of - it would have been nice, even just a few booklets or something”* (MT).

Whilst acknowledging the longer times in both ‘waiting for’ and ‘waiting in’ public hospitals, most public hospital participants were quick to defend and not question their trust in the public system, or at least the doctors and nurses working in the system. BM, a public patient, had been involved in a road accident and had been in an emergency department followed by emergency surgery. His experience was full of long ‘waiting in’ public hospitals to see a number of doctors, nurses and surgeons, but on reflection he was very philosophical regarding his treatment and expectations of the public hospital system: *“You have to think that there’s a minimum standard [in a public hospital] and that they will be able to fix you in some form and the individual talents of each one [doctors], well you’ve got no idea, simple as that”* (BM)*.* In this quote, BM trusts that public hospitals will meet a minimum standard of care and seems to reconcile the fact that patients in public hospitals have limited or no knowledge of the abilities or competence of doctors and experience longer ‘waiting in’ public hospitals, inferring a sense of pragmatic acceptance of public hospitals. BM went on to explain his lack of concern about the impact of longer ‘waiting in’ public hospitals. Although he had experience of public hospitals, he also had PHI and has used it for elective surgery and specialist appointments for his chronic conditions. In talking about the differences between ‘waiting in’ public and private hospitals, he was not concerned that public hospitals involved more waiting:

*“In a private hospital say you were to push a bell, private hospital four minutes; public hospital seven, eight ‘what’s the problem?’ If you’re really in trouble you’ve got another … and it’s not as if it’s that critical, only if you’d fallen out of bed or something in which case the others around you would surely start hollering, so I don’t see it as a big deal”* (BM).

In summary, (both public and private) had a high level of acceptance and understanding on ‘waiting in’ hospitals for emergency situations (as opposed to planned procedures), trusting that they would be taken care of: “*Okay, they might be overflowing with people but if it was a serious thing they would eventually get round to you*”. In emergency situations in public hospitals, participants talked about their internal struggle to justify the hospital that made them wait, often in pain and with little information, although they defended the system and in particular the doctors and nurses, resembling a ‘don’t bite the hand that feeds you’ philosophy. For example, *“I think we are very lucky to have this system and that we’ve got at all…they were just so busy and I do understand. There was nothing they could do and it was just going to be one of those things”.* In addition, a participant who was moved to different hospitals to deal with his fractured leg after an accident, was left in various rooms alone for hours and without adequate pain relief still managed to say *“While I was in there I got put into – they were obviously so busy and I fully respect that we have a system here and I think we’re very lucky to have this system that we’ve got at all. We come in off the street, we’re looked after and we go home and the public side of things, I do fully respect that”* (PW).

### Waiting as ‘inevitable’

It was clear that ‘waiting for’ and ‘waiting in’ public hospitals were considered inevitable by public hospital participants, but not necessarily to perceptions of untrustworthy hospitals or healthcare professionals. The inevitability was based on knowledge, through the media, of tight government budgets, increased pressure on Department of Health budgets and thus less resources for public hospitals to perform all functions for all people in a timely manner. For example, BM said:

*“The system pressures people. There’s a time pressure on everybody and that’s caused by lack of funds. That’s the ultimate driver on how we do things, isn’t it, how much money is available?....I could see that the time pressures that these guys have means that they don’t get the minute to sit and just go for thirty seconds ‘let’s just check everything’. I don’t have any grudge against them [the doctors]*” (BM).

As the above quote reiterates, the inevitability of ‘waiting for’ public hospital appointments and the time pressures on doctors and nurses due to perceptions of inadequate staffing (resulting in less time with patients and more ‘waiting in’ hospitals) does not translate into negativity, blame or distrust of doctors. It is simply seen as a ‘problem of the system’, whereby the doctors and nurses are doing their best, fulfilling the role of trustworthy professional. Complaints about waiting were almost always countered by participant’s explaining that the staff were not to blame. However, this reluctance to distrust, creates a situation where if patient are stressed or isolated or needing information, they may not feel able to ask for things since they feel responsible for adding to already overburdened hospital staff. This may not be addressed during a single hospital admission, but over time could build up and become a source of distrust. This is demonstrated in the example below where the participant is initially protective of the public health system, describing a long and painful wait in the emergency department subsequent to fracturing his leg. He was fully cognisant of the pressures on the system during his treatment and initially forgiving:

*“…I got shoved into a little room out the side, which was almost like a storage room – it was being used as a spare room basically - and every once in a while I’d press the beeper to say ‘I need this icepack refilled’ and they’d get around to doing it. It just seemed like I was in there for an eternity…. it took hours. It seemed like almost four hours and I felt like even right at the beginning there was no ice packs, there was nothing. They were just so busy and I do understand that but it was all the initial stuff. I think the fracture was a fracture, there was nothing they could do and it was just going to be one of those things* (PW).

However, when the participant had left the hospital system he described feeling very angry regarding his disjointed follow-up care and fragmented rehabilitation in the public system. He blamed the lack of follow-up for his ongoing leg problems. Although this participant expressed that he had a trust in the doctors (as general standards ensure their quality), he said that he had to organise his own rehabilitation separately from the system, because the public system ‘*will not care for you in these long-term problems*’. Thus although he was prepared to be tolerant while in the hub of care (and thus information) once outside he felt isolated, left to fend for himself, and his trust in the system had eroded.

The perceived inevitability of longer waiting lists in the public system was given as an explanation for most private hospital participants to purchase PHI. For example, DM has PHI and fully expected to receive treatment with little or no ‘waiting for’ a private hospital appointment, and his expectations had not been let down, leading to trust. He stated that he felt lucky to have PHI:

*“No matter how much money you poured into it, I think there’d always be some sort of a waiting list. You know the government just can’t do the whole – make everyone happy and just get them straight through. I just think I’m lucky that I’m able to have private insurance and can afford it and can get in early otherwise I’d have no option but to just go on the waiting list*” (DM).

Whilst not attempting to justify the inevitable ‘waiting for’ public hospitals, most participants talked about the perceived equity of access to healthcare in the public system, whereby people with greater healthcare need would have a shorter wait and people with a lower need would have a longer wait. This is akin to the concept of vertical equity, which assumes higher access to services for people in greater need for those services, and vice versa. For example, GS is a public hospital participant, but perceives a needs-based waiting system:

*“Well I don’t know much about it but my understanding is if you really need it like there’s waiting lists and you’ll be, like it’s sort of according to the need for it. So I figure if it was a major need for it I would get the operation”* (GS).

In this quote, GS trusts that someone in the public hospital system is appropriately identifying different levels of urgency and then creating a needs-based waiting list, which he is comfortable in accepting. Another participant had waited for her surgery in the public system, and recognised and trusted the needs-based approach to waiting lists. She was happy with her care, rationalising that if it had been urgent or was causing more pain, then she would have moved up the list or would have been less accepting of ‘waiting for’ her surgery:

*“I’d kind of reassured myself that he [the surgeon] did have the skills to do it, I was quite happy because he does mainly private work so I felt like I was almost getting private cover free, you know. And with the time, I may have felt differently if there’d had been a huge waiting period. It was like an ongoing issue, so it wasn’t like I urgently felt like I needed the surgery. I was like I could go on for a few months anyway so maybe that would have been different, if it was something that was causing a lot of pain”* (LI).

### Waiting as ‘bad for health’

One participant who did not have PHI articulated how she could queue jump if she had PHI and, in the quote below, stated that the process of waiting made her feel ‘inferior’:

*“It’s all time in the public system, the waiting…Trying to get in, the waiting and waiting for…Well you’re sick all the time. You think, you know ‘if I was in private health I’d be - had the operation and I’d be right again’. That’s how you feel or ‘I’d have my’ – in X hospital you have to wait a long time for scans too…. you start to feel a bit inferior again. It takes a lot to make me feel inferior now but waiting does”* (GW).

This point links to an earlier quote about people feeling ‘special’ in private hospitals, increasing their trust, whereas GW felt ‘inferior’, which could lead to lower trust in the hospital if this occurred over a longer period of time. For our participants, waiting highlighted the inequities of a society between ‘haves’ and ‘have nots’, and was seen to have negative health consequences. For many participants, the effect of waiting equated to increased stress, anxiety and worry: *“If I had been a private patient I wouldn’t have had to wait all those months worrying about God knows what I had in my throat*” (GW).

For most participants, the concerns over the negative health implications were exacerbated when it came to their children or older adults. Trust becomes an even more important issue in times of increased risk, which was the case when people talked about their children and older adults. In terms of older people, there was a perception that being on a long waiting list could not only lead to increased worry and anxiety, but to physical deterioration which would make the illness worse:

*“their condition may deteriorate that by the time they go to have the procedure they may need a bigger procedure than if it could have been attended to earlier while they were still in reasonable condition. They’re likely going to be depressed, their recovery time will be longer and if whatever’s wrong with them is deteriorating more then I think it could be a bigger procedure”* (CR).

Private hospital participants talked about their perceived responsibilities for their children, to reduce the likelihood of ‘waiting for’ hospital appointments or surgery. JM described her concern for her children and her desire that they will not have to be in a system where they will have to wait. This is not just as young children, but more a concern for them being vulnerable as they age and being in a health system with ‘*a really long queue*. This suggests that ‘waiting for hospitals is even less acceptable when people have a ‘duty of care’ for others.

*“It concerns me how vulnerable you might become as you get older in terms of an ageing population and being part of a really long queue system or not being able to get things attended to as quickly as you would like. I think that backs you into a corner to gravitate towards the private health system but at the same time the increasing costs of the private health system. If you’re unlucky or if things happen, I would be very grateful but sort of we’ve been very fortunate with my two children and my partner that no significant health issues have occurred – not to say they won’t in the future – and there’s always that unknown, that do you want that security blanket to know that if something goes wrong you will get attended to, you won’t be put in a queue*” (JM).

## Discussion

A major distinction was drawn between waiting times in regards to public and private hospitals, in terms of both ‘waiting for’ appointments/surgery and ‘waiting in’ the hospital as a patient. ‘Waiting for’ hospital appointments were seen as very different in public (longer waiting times) as opposed to private hospitals (shorter waiting times). In the public system, participants felt that longer waiting times were unavoidable, given the finite government money and other government priority spending decisions, thereby not leading to an overriding distrust of public hospitals. Therefore, longer times ‘waiting for’ hospital appointments and elective surgery became accepted (although not necessarily liked), trusting that public hospitals were doing they best they could within the current financial climate. This finding is similar to those in a qualitative study in Canada [1] which found that some patients waiting for elective surgery had a ‘passive acceptance’ of waiting for surgery, given the shortage of doctors and finite government budget, some even regarded waiting as a ‘part of life’. In our study, an equity lens was visible to participants who were ‘waiting for’ public hospitals, whereby there was a perception that generally people with higher need got ‘bumped up the queue’ and this was seen as equitable and acceptable. This ‘vertical equity’ [39] is the bedrock of most publically-funded healthcare systems, whereby people in higher need get access to services more than people with lower need. This perspective is in stark contrast to what might be evident in a neoliberal political climate whereby individualistic approaches to health are focused around individual and proactive risk minimization [26].

Given the inevitability and resigned acceptance of ‘waiting for’ public hospitals, and to a certain extent the equity lens, participants did not blame or distrust doctors or nurses, since the ‘fault’ was levelled at the system level, within which doctors and nurses worked. In this way, participants still felt trusting of doctors and nurses in public hospitals, because they were perceived to be doing their best in an under-funded system and therefore exonerated from blame. Nevertheless, longer waiting times both ‘for’ and ‘in’ public hospitals were perceived as most problematic for children and older people – whereby the responsibility was taken on by the participant for their child or older relative. Longer times ‘waiting for’ public hospitals were perceived to increase stress and anxiety, and with older people, potentially make their health condition worse (e.g. make their ailing hip worse whilst waiting for a hip replacement). The stated link by participants between longer waiting times leading to poorer mental and physical health outcomes results in a questioning in trust of the system. Whilst other studies have also found the link between longer waiting times and perceived poorer physical and mental health [1–4], our analysis highlights the ways in which some patients begin to question their trust in the system and the doctors and nurses within the hospitals.

In terms of ‘waiting in’ public hospitals, participants talked about their perceptions of lack of nurses, the higher workload for nurses and the ‘chaotic’ systems, which were based on their experiences as patients and carers in public hospitals. The experiences of busy, rushed and over-worked nurses was seen to lead directly to their experiences of longer waiting times in emergency departments and on hospital wards. Experiences of having less time with the nurses and longer waits to be seen by nurses (e.g. after ringing the buzzer at the side of the bed) was compared with the private hospitals which were perceived to have more nurses per patient, allowing nurses to spend more time with each patient and patients not having to wait as long to be seen (e.g. after ringing the buzzer). Nevertheless, although ‘waiting in’ public hospitals was seen as more of a problem and caused more stress than in private hospitals, it did not affect trust in nurses because they were still perceived to be ‘doing their best’ within the given constraints, a key ingredient in a trusting relationship.

One way of understanding and interpreting our key findings, is through literature on institutional trust [5, 6, 9, 10, 22, 23, 36, 40–42]. Within the literature on institutional trust, two different types of ‘trust norms’ have been proposed [43] – ‘exchange trust norms’ and ‘communal trust norms’. *Exchange trust norms* focus on expectations of consistency, cautious decision-making, high service standards, and are usually expected of institutions such as banks and the judiciary. For example, if banks are shown to exhibit these attributes, they are seen as trustworthy. *Communal trust norms* include additional expectations of flexibility, adaptability and helpfulness, which are usually expectations of non-government organisations and welfare organisations. Our data seem to suggest that patients have expectations of public hospitals similar to exchange trust norms, a kind of base-level set of expectations that make them trustworthy. Public patients have experienced longer ‘waiting in’ and ‘waiting for’ public hospitals, and they have come to expect this situation, justifying it through reduced government funding, an ‘equity lens’ and over-worked healthcare professionals. In this way, public patients expect a basic level of exchange trust norms, including a minimum accepted standard of safe and appropriate care and waiting times appropriate to healthcare need. Public patients would like the additional communal trust norms expected of private hospitals, such as reduced waiting times and more time with healthcare professionals, but recognise the inevitability of the current situation within the context of government funding and priorities. However, public hospitals, from our data, are not necessarily expected to exhibit these additional attributes of communal trust norms. In this way, patients can still trust public hospitals because they adhere to their exchange trust norms, whereas trust in private hospitals has the added expectation of communal trust norms. Private patients have come to expect reduced waiting times and more time spent with healthcare professionals, and judge their trust in private hospitals with these additional communal trust norms. We suggest that these expectations could be added to the list of expectations for communal trust norms in private hospitals. Private patients also use these communal trust norms to judge public hospitals, and since public hospitals do not meet these expectations, they have lower trust in public hospitals. However, we suggest that public hospitals should not necessarily be judged by communal trust norms, and instead by exchange trust norms. We certainly do not suggest that public hospitals should have a lower standard of care, but that exchange trust norms should be a minimum standard of care expected of public hospitals, making them trustworthy, and the ‘added benefits’ accrued by communal trust norms should not be used to judge the trustworthiness of public hospitals. At this stage, these are tentative suggestions, which seem to us, very worthy of further research.

## Conclusions

As demonstrated by our data, research on (dis)trust in public hospitals in Australia is urgently needed. Medicare, since its inception, has been under media scrutiny and negative media stories about ‘problems’ associated with public hospitals have been shown to decrease public trust [29]. Successive Liberal governments have placed increasing emphasis on privatisation. Government rebates to facilitate purchase of PHI and additional Medicare charges for high income-earners who do not take out PHI are explicitly aimed at building the private health market. In 2014, the incoming Liberal Premier of New South Wales stated his intention to privatise public hospitals and services, arguing that private companies would “improve healthcare” [44]. In 2016, the Prime Minster and Federal Minster for Health announced that they are considering potential further privatisation of public health payment systems (currently undertaken by Medicare), suggesting savings of around $50 billion, while asserting such measures will improve services [45]. In this context of increasing calls for further privatisation, understanding the impact of waiting times for public patients is of central importance since negative perceptions of public hospitals fuelled through the media (potentially leading to mistrust and poorer patient outcomes) may have a much more profound effect than positive ones (potentially maintaining trust), with some authors suggesting that “trust comes on foot and goes away on horseback” [46].
